# Investigation of Rheological Characteristics of Thermosensitive Nasal In Situ Gels Based on P407 and Their Effect on Spray Pattern

**DOI:** 10.3390/gels11100841

**Published:** 2025-10-21

**Authors:** Natalia Menshutina, Vladislav Derkach, Elizaveta Mokhova, Mariia Gordienko

**Affiliations:** The Department of Chemical and Pharmaceutical Engineering, Mendeleev University of Chemical Technology of Russia (MUCTR), Moscow 125047, Russia; derkach.v.s@muctr.ru (V.D.); mokhova.e.k@muctr.ru (E.M.); gordienko.m.g@muctr.ru (M.G.)

**Keywords:** nasal delivery, spray, poloxamer, mucoadhesive polymers, in situ gels, rheology, spray pattern, mathematical modeling

## Abstract

This article presents the results of a study on the rheological characteristics of in situ thermosensitive nasal gels based on poloxamer 407 (P407) and their effect on spray angle. The development of new drug delivery systems based on in situ thermosensitive gels can overcome several shortcomings of traditional nasal sprays associated with mucociliary clearance and low mucoadhesion. Using the cold method, samples based on P407 were prepared in pure form, in combination with poloxamer 188 (P188), and with the addition of several mucoadhesive polymers: chitosan, sodium alginate, and hydroxypropyl methylcellulose (HPMC). Analytical studies were carried out for all obtained samples, which showed that the gelling temperature (T_sol–gel_) of compositions with P407 was inversely dependent on its concentration, decreasing from 32.71 °C to 24.63 °C. The addition of hydrophilic P188 increased T_sol–gel_. The addition of mucoadhesive polymers had varying effects on T_sol–gel_: chitosan and HPMC increased the temperature, while sodium alginate decreased it. The addition of mucoadhesive polymers significantly affected the viscosity of the formulations; for example, the addition of sodium alginate resulted in a fivefold increase, making the formulations unsuitable for spraying. A study of the spray angles of T_sol–gel_ samples in the range of 27–31 °C using the SprayVIEW measuring system revealed an inverse relationship between the viscosity of the formulations and the spray angle. A mathematical model of the solution droplet trajectory was presented, enabling the spray angle to be predicted depending on the formulation composition. The relative error of the computational experiments did not exceed 10%. This approach has the potential to reduce the number of full-scale experiments, and consequently their cost.

## 1. Introduction

The intranasal route of administration is one of the most promising non-invasive methods of delivering drugs into the human body [1]. The nasal mucosa is highly permeable and has a well-developed network of blood vessels, ensuring rapid drug delivery into the bloodstream [2,3]. Furthermore, the nasal route allows for targeted drug delivery to the central nervous system via the olfactory and trigeminal nerves, which are directly connected to the brain, enabling the drug to cross the blood–brain barrier [4,5,6]. The intranasal route of administration allows bypassing of the aggressive environment of the gastrointestinal tract and the “first-pass effect” through the liver barrier [7,8]. However, the key disadvantages that limit the effectiveness of nasal preparations are mucociliary clearance and dripping of the dosage form into the oropharynx, which leads to low bioavailability of the active substances [9]. A promising approach to overcome these disadvantages is the use of heat-sensitive in situ gels, which are liquid at room temperature and form a gel upon contact with the nasal mucosa (32–35 °C) [10,11]. In addition, in situ gelling systems offer a number of advantages including high drug loading efficiency, sustained release profile, improved nasal absorption, and longer residence time as well as reduced dosing frequency and improved patient compliance [12,13].

Among the various polymers capable of forming thermosensitive gels in situ, P407 is one of the most studied due to its biocompatibility and non-toxicity [14]. This triblock copolymer PEO_100_-PPO_65_-PEO_100_ has an amphiphilic nature, due to which it exhibits thermosensitivity [15]. At low temperatures, P407 molecules are present in solution as monomers or loosely bound aggregates, which gives the solution a liquid, low-viscosity state [16]. With increasing temperature, dehydration of the hydrophobic polypropylene oxide (PPO) block occurs, which initiates the process of the self-assembly of molecules into micelles with a hydrophobic PPO core and a hydrophilic polyethylene oxide (PEO) shell. When the poloxamer concentration is high enough and the temperature reaches the sol–gel transition point, the micelles begin to tightly pack and form a three-dimensional network structure—a gel [17].

Adjusting the concentration of P407 allows for the regulation of the gelation temperature; however, auxiliary components are often added to achieve optimal characteristics [18]. Among the auxiliary components used to regulate the in situ gelation temperature of P407-based gels, P188 has received particular attention [19]. This polymer, which has a triblock structure of PEO_76_-PPO_29_-PEO_76_, is characterized by higher hydrophilicity compared with P407 due to the increased ratio of hydrophilic PEO blocks to hydrophobic PPO [20]. The addition of P188 allows for an increase in the gelation temperature of the system due to changes in the micellar structure and the slowing down of micelle aggregation, which expands the possibilities for fine-tuning the temperature properties of the gels [21]. Other auxiliary components for regulating the gelling temperature and increasing the mucoadhesive properties are mucoadhesive polymers such as chitosan, HPMC, and sodium alginate. Chitosan is a natural cationic polysaccharide that improves gel adhesion to the nasal mucosa through electrostatic interactions between its positively charged amino groups and the negatively charged mucin components of mucus [22]. HPMC is a polysaccharide derivative of cellulose, widely used as a thickener and stabilizer; its ability to form hydrogen bonds with mucin on the surface of mucous membranes enhances gel adhesion and prolongs its residence time at the injection site [23]. Sodium alginate is an anionic polysaccharide that forms hydrogels when interacting with divalent cations; it also has high biocompatibility and increased mucoadhesion [24]. The selected polymers included in the nasal thermosensitive in situ gel—poloxamer 188, chitosan, HPMC and sodium alginate—are biocompatible and safe additives for nasal drug delivery systems [25,26,27,28,29].

Currently, a significant amount of scientific data have been accumulated confirming the potential of developing complex systems based on P407 in combination with various polymer additives. Such complex systems not only allow for the optimal sol–gel transition temperature range for practical use to be achieved (27–31 °C), but also enhance mucoadhesion, prolong the retention time on the mucous membrane, and ensure controlled release of the drug, which together increases the efficiency and safety of intranasal delivery [30,31,32,33,34]. In the work of Fathalla Z. et al., it was demonstrated that the addition of chitosan to poloxamer 407 solutions reduces the gelation time and enhances the mucoadhesive properties of the system [35]. In the work of Talasaz A.H. and co-authors, they noted that the addition of HPMC to a P407 solution allowed for the concentration of P407 required for in situ gel formation to be reduced from 18 wt% to 10 wt% [36].

Developing nasal formulations is challenging because the rheological properties of the formulations directly impact the spray angle, droplet size, and uniformity of drug distribution in the nasal mucosa [37]. To select formulations and study the effect of viscosity on spray angle in full-scale experiments, unique and expensive equipment is used, which significantly increases the cost of developing nasal formulations. One approach to reducing the development costs is the use of modern mathematical modeling methods, such as computational fluid dynamics, which enable a comprehensive study of the dependence of spray angle on the rheological properties and reduce the formulation selection time [38,39].

The aim of this study was to investigate the effects of P407 concentration, its admixture with P188, and the addition of mucoadhesive polymers (chitosan, HPMC, sodium alginate) on the rheological properties (loss modulus, storage modulus, viscosity), gelation temperature, and spray pattern characteristics of nasal in situ temperature-sensitive gels. The data obtained will enable the selection of gel formulations to achieve a balance between ease of administration, delivery efficiency, and drug retention in the nasal cavity.

## 2. Results and Discussion

### 2.1. Rheological Properties of Hydrogels Based on P407

The optimal formula for a thermosensitive in situ gel should have a T_sol–gel_ temperature above room temperature (20–25 °C) and below the temperature of the nasal mucosa (32–35 °C) [40]. With this T_sol–gel_ range (29 ± 2 °C), solutions can be directly sprayed, and at this T_sol–gel_, gel formation will occur fairly quickly upon contact with the nasal mucosa, which will theoretically reduce the percentage of drug loss due to dripping into the oropharynx.

**Figure 1:**
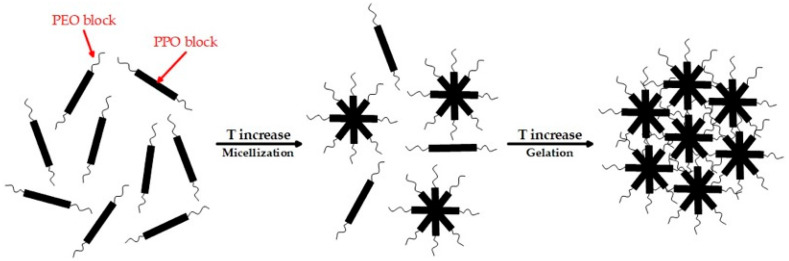
Schematic representation of micellization followed by the gelation of P407.

Based on the specific temperature range of the sol-to-gel transition (27–31 °C), P407 was chosen as the main gelling agent. Moreover, according to the literature, P407 is the least toxic of the commercially available poloxamers [41,42,43]. P407 is an amphiphilic block copolymer consisting of a central hydrophobic block of PPO surrounded by two hydrophilic blocks of PEO [44]. At temperatures below the sol-to-gel transition point (T_sol–gel_), P407 molecules exist primarily as monomers or loosely bound aggregates in solution, resulting in a low-viscosity liquid state of the system (sol). An increase in temperature causes dehydration of the hydrophobic PPO blocks, initiating the self-assembly of P407 molecules into micelles with a hydrophobic PPO core and a hydrophilic PEO shell [45]. With a further increase in temperature and a sufficient polymer concentration, these micelles are tightly packed and arranged into ordered structures [46]. This process leads to the formation of a three-dimensional network, which causes the system to transition to a gel state (Figure 1).

**Figure 2:**
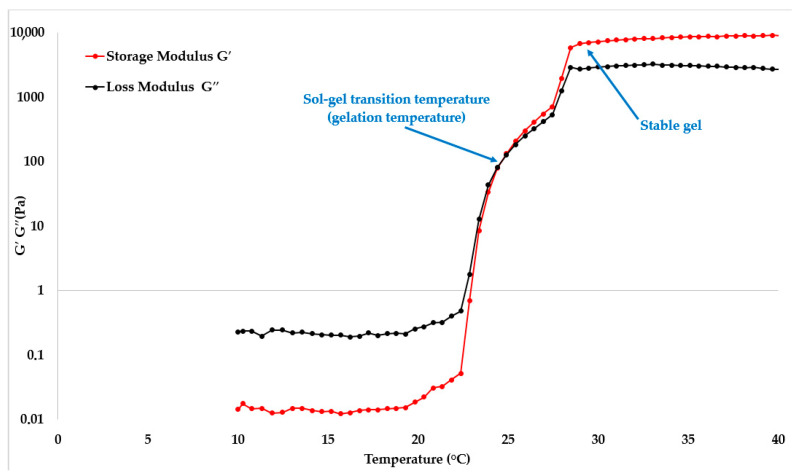
Plot of the storage modulus G′ and loss modulus G″ as a function of temperature in situ for a gel with a P407 concentration (20 wt%). Blue arrows indicate the sol–gel transition temperature, where G′ and G″ are equal, and the formation of a stable gel.

To determine the T_sol–gel_, graphs of the storage modulus (G′) and loss modulus (G″) versus temperature were constructed for each sample. The storage modulus is proportional to the energy stored and returned during vibrations, while the loss modulus is proportional to the energy lost due to friction [47]. Therefore, in predominantly elastic solids, G′ > G″, and in viscous liquids, G″ > G′. The T_sol–gel_ can be defined as the intersection point of G′ and G″ (Figure 2).

To study the in situ thermosensitive properties of the P407-based gel, the effect of P407 concentration on the gelation temperature of the system was studied (Table 1).

**Figure 3:**
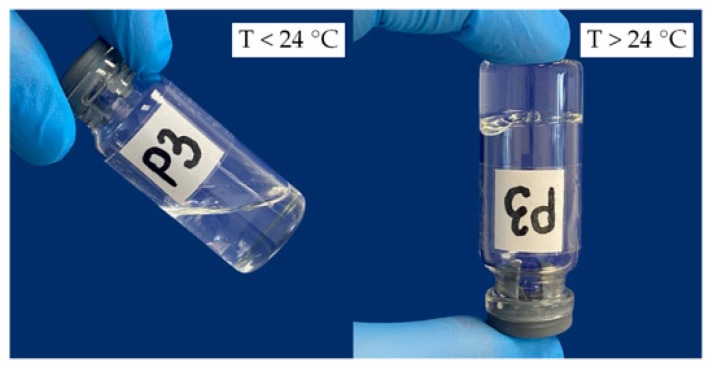
Sol–gel transition process using sample P3 as an example.

It was found that the nasal in situ temperature-sensitive gels remained fluid at temperatures below 24 °C, but rapidly gelled after heating above 24 °C (Figure 3).

**Figure 4:**
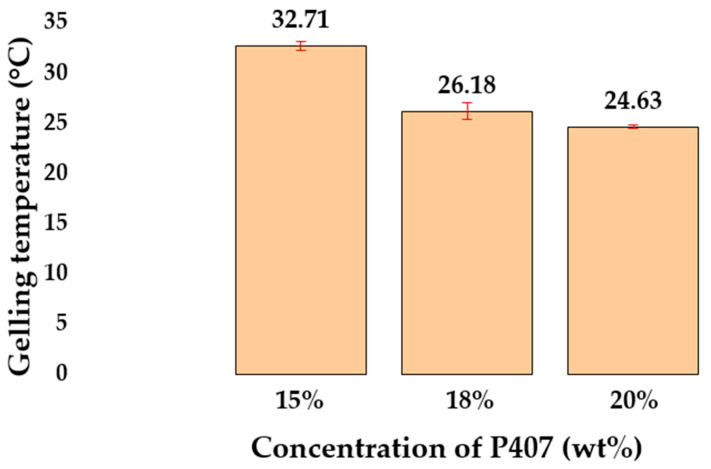
Average in situ gelation temperature of the P407 gel at different concentrations.

Based on the experimental results obtained, a pronounced dependence of the T_sol–gel_ on concentration was revealed: with an increase in the content of P407, a decrease in the T_sol–gel_ was observed from 32.71 ± 0.47 °C to 24.63 ± 0.14 °C (Figure 4). The obtained T_sol–gel_ of the P407 solutions are consistent with the data of other researchers [21,48,49]

The G′ values at the nasal mucosa temperature (35 °C) for samples P1, P2, and P3 were 209.15 ± 23.71, 5501.83 ± 349.47, and 8581.36 ± 236.18 Pa, respectively. A pronounced tendency for G′ to increase with increasing polymer concentration was observed. The literature notes that in situ gels with a high G′ value are capable of forming a strong gel network on the surface of the nasal mucosa, which promotes longer retention of the drug [50,51]. However, too high a G′ may lead to an excessive increase in viscosity, which hinders uniform spraying and distribution of the drug on the nasal mucosa, and may also cause patient discomfort during use. Despite the relatively low G′ values for samples P2 and P3, their weaker gel network is expected to result in higher drug diffusion than dense gels with a high storage modulus, which is particularly important in the context of intranasal delivery. The resulting dependence can be explained by the fact that increasing the P407 concentration in the solution increases the number of polymer chains, which enhances the hydrophobic interactions between the PPO blocks, facilitating micelle aggregation at lower temperatures. Higher concentrations enhance interactions between micelles, leading to the formation of a gel network at lower temperatures, thereby lowering the gelation temperature and increasing the storage modulus.

Based on the obtained gelation data, compositions P2 and P3 were selected for further study due to their suitable gelation temperatures (T_sol–gel_ = 27 ± 3 °C).

### 2.2. Rheological Properties of Hydrogels Based on P407 and P188

To improve the in situ performance of the P407-based gels, P188, which is often used for more precise tuning of the gelation temperature [52,53,54], was selected. For this purpose, composite mixtures of P407 and P188 were studied in various mass ratios at a constant total concentration of poloxamers (P407/P188 = 9/1 and 8/2)—Table 2.

**Figure 5:**
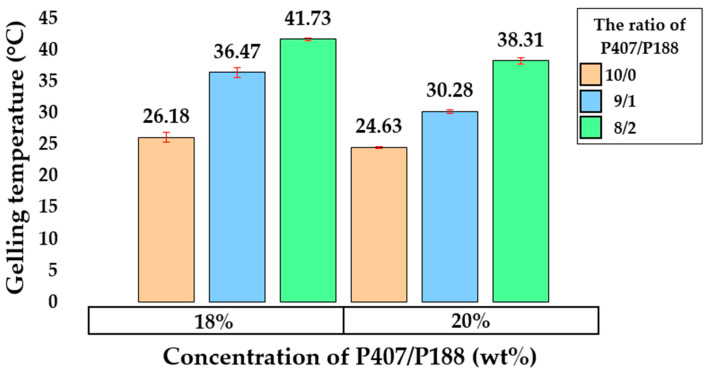
Average in situ gelation temperature of a P407/188 gel at different ratios. Beige bars: P407 only; light blue bars: poloxamers 407/188 = 9/1; light green bars: poloxamers 407/188 = 8/2.

As can be seen in Figure 5, a trend was observed indicating that P188 increases the T_sol–gel_ regardless of the total poloxamer concentration. This dependence is consistent with data from other researchers [55,56,57,58]. Of the obtained P407/P188 composite mixtures, sample PP3 was the most suitable for nasal delivery, as its T_sol–gel_ was 30.28 ± 0.33 °C. The addition of P188 to P407 solutions resulted in a significant decrease in G′ for all samples, the resulting dependence being consistent with previously published data [59].

Many studies have demonstrated the dependence of the gelling properties of poloxamers on the ratio of hydrophobic PPO to hydrophilic PEO blocks in the polymer chain structure [59,60]. Based on the literature data, P188 is characterized by a higher PEO/PPO ratio compared with P407, which makes it a more hydrophilic polymer.

The more hydrophilic properties of P188 result in a higher critical micelle temperature than P407. This can be explained by the formation of a significant number of hydrogen bonds between the hydrophilic PEO blocks and water molecules. Due to the increased number of hydrogen bonds, additional energy is required to break them, leading to an increase in the sol–gel transition temperature [56,59,61]. This is why, when mixing P407 and P188, an increase in the gelation temperature and a decrease in the storage modulus of the composite mixture were observed.

### 2.3. Rheological Properties of Hydrogels Based on P407 and P188 with the Addition of Mucoadhesive Polymers

Other polymers were added to P407-based gels to increase the viscosity and mucoadhesiveness, which should facilitate greater control over drug release. The mucoadhesive polymers selected were the cationic polymer chitosan, the neutral polymer HPMC, and the anionic polymer sodium alginate. The effect of the mucoadhesive polymers on the gelation temperature depends on the nature of these polymers and their concentration in the samples (Table 3).

Upon adding 0.2 wt% chitosan to 18 and 20 wt% P407 solutions, an increase in the T_sol–gel_ by an average of ~2 °C was observed compared with the initial solutions. With a further increase in the chitosan mass (0.6 wt%), the T_sol–gel_ values decreased to the initial values for the 18 and 20 wt% P407 solutions. Samples PC1–PC6 also showed a significant weakening of the gel network, with their G’ values being several times lower than those for solutions containing only P407. Similar results were obtained by Laquintana V. et al., in which the addition of 1 wt% acetic acid to a 16 wt% P407 solution resulted in a two-order-of-magnitude decrease in G’ [62]. The increase in T_sol–gel_ and weakening of the gel network can be explained by the presence of acetic acid, which is used for the direct dissolution of chitosan. Acetic acid weakens the hydrophobic interactions between the PPO blocks, which hinders aggregation and micelle packing, thereby increasing the T_sol–gel_ and decreasing the G′ [62,63].

In general, formulations containing HPMC exhibited an increase in the T_sol–gel_ of P407 solutions, which may be due to a disruption of the micelle packing mechanism. This effect is due to the hydrophilic nature of HPMC, which increases the overall hydrophilicity of the mixture. As a result, the formation and packing of poloxamer micelles, which mediate the sol-to-gel transition, are slowed, requiring a higher temperature for the development of the gel network [64,65]. Despite the slight decrease in T_sol–gel_, the addition of HPMC also resulted in an increase in G′ at 35 °C, which in turn reflects the formation of a denser gel network.

Although the addition of sodium alginate reduced the gelation temperature and increased the storage modulus of the 18 wt%P407 solutions, the resulting solutions were highly viscous even at room temperature, making these compositions unsuitable for dispensing with dispensing devices. For 20 wt% wt. solutions of P407, sodium alginate increased the G′ value but had virtually no effect on T_sol–gel_. The PA4–6 samples also had high viscosity at room temperature, which hindered their sprayability.

### 2.4. Results of Viscosity and Density Studies

To model the spray angle, the physicochemical properties of thermosensitive nasal in situ gels, specifically viscosity and density, were studied. The results (Table 4) demonstrate that the density values of all of the studied samples lay within a narrow range of 0.990–1.020 g/cm^3^, which is typical for concentrated aqueous solutions of P407.

Increasing the concentration of P407 resulted in a slight increase in density from 1.003 ± 0.004 to 1.007 ± 0.003 g/cm^3^. The effect of polymer additives (such as P188, chitosan, HPMC, and sodium alginate) on density was insignificant. With the addition of P188, a slight decrease (less than 1.000 g/cm^3^) was observed, while in the presence of HPMC and sodium alginate, a slight increase was observed, which may be due to an increase in the mass fraction of dissolved mucoadhesive polymers and the formation of denser polymer networks.

The viscosity of thermosensitive nasal in situ gels depends significantly on the concentration of P407, which directly influences the formation of polymer micelles. A pronounced concentration dependence was observed: increasing the mass fraction of P407 from 15% to 20% led to a more than twofold increase in viscosity, from 10.44 mPa·s to 37.71 mPa·s. This sudden increase may indicate the onset of a more compact gel structure due to the increased number and intertwining of hydrophobic PPO blocks.

The introduction of polymers into the in situ composition of P407-based gels has a significant effect on their viscosity. The presence of P188 in an 18–20 wt% P407 solution led to a decrease in viscosity, which can be explained by its lower ability to form a strong gel structure and the predominance of hydrophilic blocks, which disrupt the integrity of the micellar network. Conversely, the addition of chitosan leads to a marked increase in viscosity. Increasing its concentration in the system from 0.2 wt% to 0.6 wt% led to an almost fivefold increase in viscosity. Thus, the viscosity of an 18 wt% P407 solution with 0.2 wt% chitosan is approximately 35 mPa·s, while in a solution with 0.6 wt% chitosan, this figure exceeds 170 mPa·s. This effect is due to the polycationic nature of chitosan, which enters into intermolecular interaction with poloxamer micelles, significantly increasing the density and elasticity of the gel network.

HPMC exhibited a concentration-dependent increase in the viscosity of the composites in the studied range (0.5–2 wt%). Thus, in a system with 18 wt% P407, an increase in the HPMC concentration from 0.5 wt% to 2 wt% led to a slight increase in viscosity from 32.31 ± 2.99 to 48.16 ± 3.76 mPa·s. This increase in viscosity is due to the fact that HPMC has a large number of hydroxyl groups, which actively bind water molecules, forming a stable viscous layer surrounding the liquid. This leads to an increase in the volume of the hydrated shell of the polymer chains, which, in turn, significantly increases the viscosity.

Sodium alginate had the most significant effect on the rheological properties. Adding 0.2–0.6 wt% alginate to an 18 wt% P407 solution caused a sharp increase in viscosity from 99 to 457 mPa·s. This pronounced effect is explained by alginate’s ability to form highly durable and viscous polymer networks, which significantly increase the mechanical strength and overall viscosity of the gel matrix.

The data obtained allow for the effective selection of formulations and regulation of the viscosity properties of thermosensitive nasal gels, ensuring their stability, comfortable administration, and retention on the mucous membrane.

### 2.5. SprayVIEW Spray Pattern Study Results

The criterion for selecting the leading samples was the gelation temperature, which should be in the range from 27 to 31 °C. Therefore, the following samples were selected for the spray angle studies: P2, PP3, PC1, PC2, PC4, PH1, and PH2.

The spray angle of the nasal spray was determined by passing a laser beam axially through the cone-shaped spray. Images of the spray’s axial cross-section were captured by a camera during spraying and then processed using Viota software (Version: 8.2.0).

The obtained data demonstrated an inverse relationship between the viscosity of the solutions and the spray angle (Table 5).

**Figure 6:**
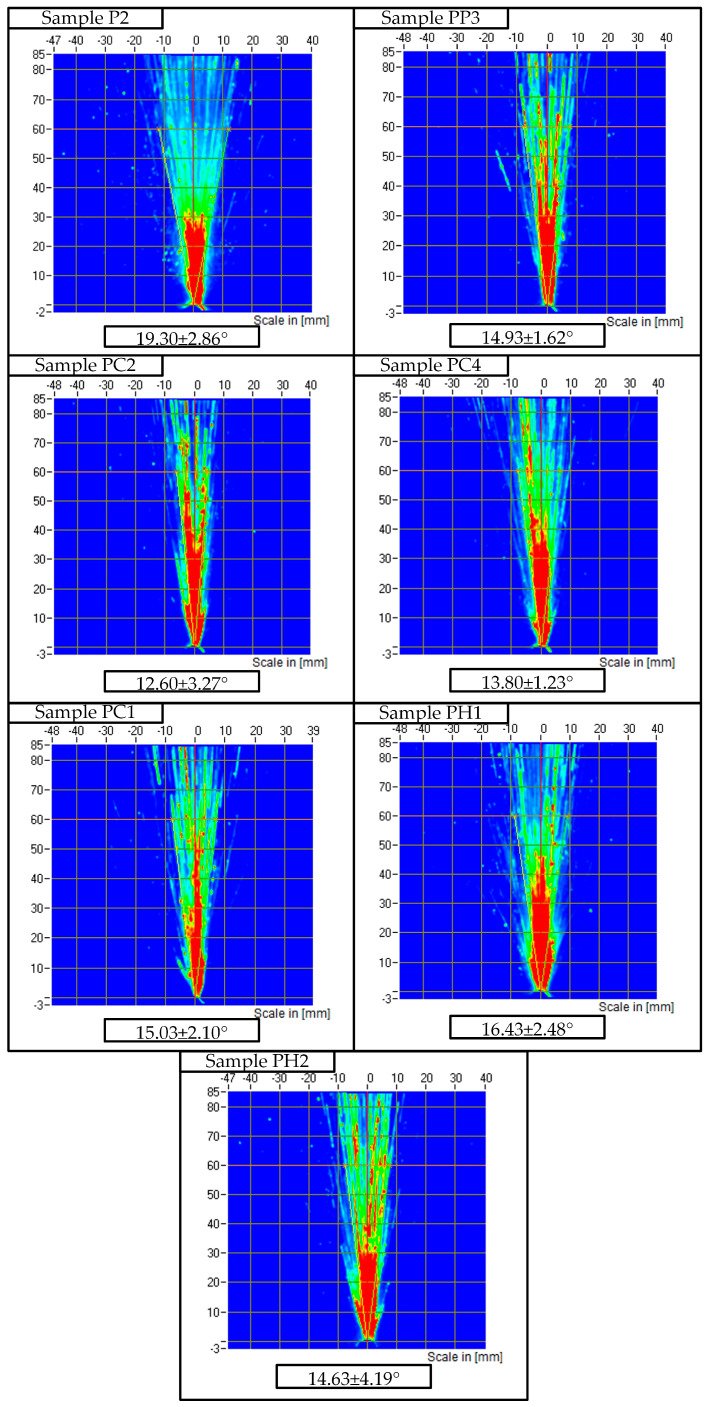
Spray patterns and spray angle values for samples P2, PP3, PC1, PC2, PC4, PH1, and PH2.

The initial 18 wt% solution of P407 with a viscosity of 18.22 ± 0.46 mPa·s produced a fairly wide spray angle of 19.3 ± 2.86° (Figure 6). The addition of P188 to a 20 wt% solution of P407 narrowed the spray angle to approximately 14.93 ± 1.62°.

The addition of chitosan (0.2–0.4 wt%) to a P407 solution increased the viscosity of the system and led to a narrowing of the spray angle to 12.60–15.03°, which is associated with decreased flowability and increased resistance of the sprayed liquid to dispersion. Similarly, HPMC at concentrations of 0.5–1 wt% led to an increase in viscosity and a decrease in the spray angle to 14.63–16.43°.

Thus, the presented results demonstrate a pattern: an increase in the viscosity of thermosensitive gels based on P407 in the presence of various polymer additives is accompanied by a decrease in the spray angle, limiting the width of the spray zones during intranasal use.

### 2.6. Results of Mathematical Modeling of the Spray Torch

**Figure 7:**
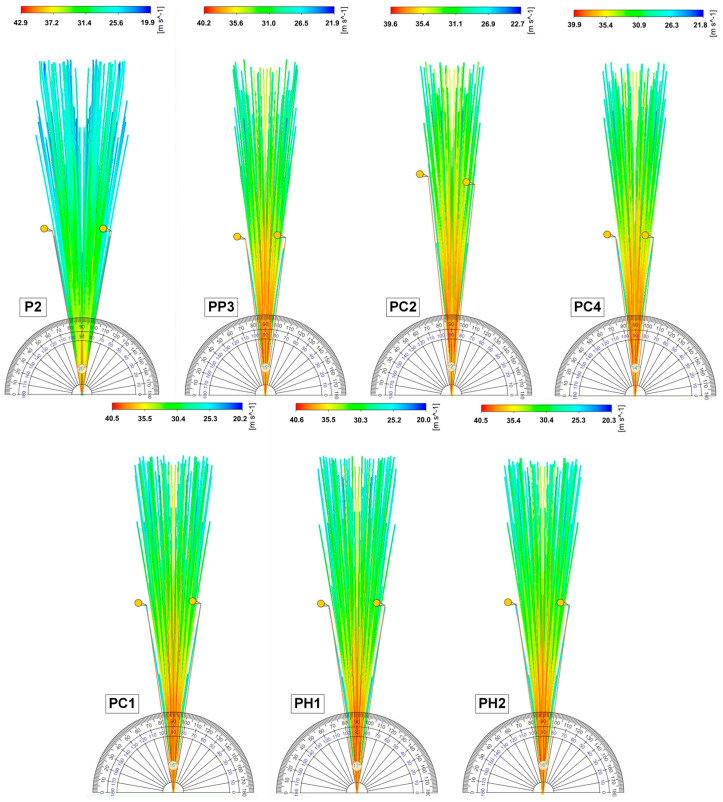
Streamlines reflecting the spray direction and speed.

In this work, a series of computational experiments were carried out to predict the spray torch for the leading compositions: P2, PP3, PC1, PC2, PC4, PH1, and PH2 (Figure 7) using the mathematical model given in Section 4.7.

The spray angle was measured for each composition using a digital protractor.

**Figure 8:**
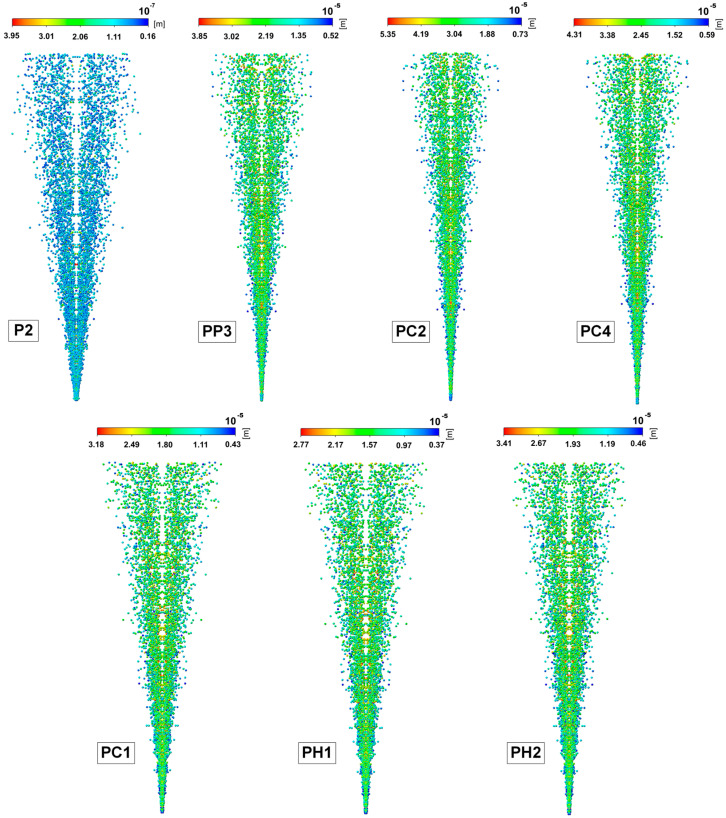
Results of the droplet size prediction for leading formulations at the outlet of a dispersing device.

Figure 8 shows the results of calculating the diameter of the droplets that formed at the outlet of the dispersing device.

Based on the obtained results of the computational experiments, it was concluded that the spray angle is highly dependent on the viscosity of the formulations: the higher the viscosity, the smaller the spray angle. These results are consistent with the experimental data obtained using the SprayVIEW measuring system. The highest droplet velocity was observed in the central part of the spray cone, while the velocity decreased at the periphery. The droplet size also depends on the viscosity and increases with increasing viscosity. For formulation P2, which had the largest spray angle (20°) and the lowest viscosity of 18.22 mPa·s, the maximum droplet size was 3.95·10^−7^ m, while for formulation PC2, which had the smallest spray angle (12°) and the highest viscosity of 68.13 mPa·s, the maximum droplet size was 5.35·10^−5^ m.

The relative error in calculating the spray angle was determined using Equation (1):

(1) $$\delta =\frac{\left|A_{c}-A_{e}\right|}{A_{e}}\cdot 100\%,$$

where *δ* is the relative calculation error, %; *A_c_* is the spray angle according to the model, °; *A_e_* is the spray angle according to the experiment, °.

The results of calculating the relative error of the computational experiments are given in Table 6.

The proposed model for predicting the characteristics of the spray cones of liquid nasal dosage forms quite reliably reproduced the shape and angle of the spray cone, which was confirmed by a low relative error of <10%.

## 3. Conclusions

The study established relationships between the composition, rheological properties, and spray angle of thermosensitive nasal in situ gels. The gelation temperature of P407-based compositions was inversely related to its concentration; that is, with an increase in concentration from 15 to 20 wt%, the temperature decreased from 32.71 ± 0.47 °C to 24.63 ± 0.14 °C. This phenomenon is associated with an increase in the number of hydrophobic PPO blocks, which leads to micelle aggregation at lower temperatures. The addition of P188 to P407 solutions resulted in an increase in the gelation temperature due to a higher critical micelle temperature because of the hydrophilic nature of P188. The addition of mucoadhesive polymers such as chitosan and HPMC resulted in a slight increase in the T_sol–gel_. The increase in T_sol–gel_ for chitosan may be due to the presence of acetic acid, which increases the number of hydrogen bonds, and for HPMC, to an increase in the overall hydrophilicity of the composition. The addition of sodium alginate slightly decreased the T_sol–gel_ but significantly increased the viscosity of the system. The presence of 0.6 wt% alginate in an 18 wt% P407 solution caused a sharp increase in viscosity from 18.22 ± 0.46 to 457.14 ± 51.19 mPa·s.

Since in situ heat-sensitive gels must be liquid at room temperature (20–25 °C) but gel upon contact with the nasal mucosa (32–35 °C), the leading formulations were selected based on the established gelation temperature range of 29 ± 2 °C. Therefore, the following samples were selected for spray angle studies: P2, PP3, PC1, PC2, PC4, PH1, and PH2. It was found that with increasing viscosity of the compositions, the spray angle decreased. The largest spray angle was observed for composition P2 (19.3 ± 2.86°) with a viscosity of 18.22 ± 0.46 mPa·s. Sample PC2, with the highest viscosity of 68.13 ± 6.04 mPa·s, formed the narrowest spray fan of all samples (12.6 ± 3.27°). Based on the conducted studies, the PP3 sample was selected as the leading composition (20 wt% P407/P188 in a ratio of 9:1), since it best met all the key requirements for thermosensitive nasal in situ gels: optimal gelling temperature (30.28 ± 0.33 °C) and relatively low viscosity (41.16 ± 1.35 mPa·s at 20 °C), which can ensure rapid gelling on the nasal mucosa and will not hinder its spraying at room temperature. A study on the SprayVIEW system showed that the PP3 sample formed a spray fan angle of 14.93 ± 1.62°. This angle is narrow enough to ensure targeted delivery and prevent dripping into the oropharynx, but not too small that it could result in insufficient coverage of the nasal mucosa.

The obtained experimental data on the study of spray torches of nasal compositions were consistent with the computational experiments, the relative error of which did not exceed 10%.

The combination of experimental studies and mathematical modeling methods is a promising approach for the accelerated development of new nasal in situ temperature-sensitive gels. Despite the extensive literature on the development of in situ thermosensitive gels for nasal delivery, the strength of this study lies in its investigation of the influence of rheological properties on the spray angle as well as its prediction using mathematical modeling methods. The results obtained in this study of spray shape depending on the in situ rheological properties of nasal thermosensitive gels will be used in further in vitro studies on a 3D model of the human nasal cavity to determine the irrigation area. A combination of mathematical modeling methods for the movement and deposition of nasal gel particles in the human nasal cavity with experimental studies on a 3D model of the nasal cavity is also of interest for future work. This approach will, firstly, confirm the adequacy of the mathematical model and, secondly, reduce the number of experimental studies and the time required to select leading formulations.

## 4. Materials and Methods

### 4.1. Substances Used

To develop thermosensitive in situ gels, P407 (Sigma-Aldrich, St. Louis, MO, USA) and the following excipients were used in the experiment: P188 (Sigma-Aldrich, USA), HPMC Headcel 60HD15 (Shandong Head Co., Ltd., Zibo, China), sodium alginate (Sigma-Aldrich, USA), chitosan (Sisco Research Laboratories, Mumbai, Maharashtra, India).

### 4.2. Method for Obtaining Gels

Nasal thermosensitive in situ gels were prepared using the cold method [66,67,68]. Purified water for gel preparation was cooled at 4 °C overnight. The required amount of dry P407 powder (15, 18, and 20 wt%) was then dissolved in 10 mL of cooled purified water and stirred for 30 min on a magnetic stirrer at 200 rpm (IKA, Staufen, Germany). The samples were then cooled overnight at 4–5 °C to obtain a clear solution.

P188 was added to the resulting P407 hydrogels in various ratios (9:1 and 8:2) and weighed portions of mucoadhesive polymers. Chitosan (0.2–0.6 wt%), HPMC (0.5–2 wt%), and sodium alginate (0.2–0.6 wt%) were selected as the mucoadhesive polymers. Table 7 presents the compositions of the resulting nasal thermosensitive in situ gels.

For the compositions given in Table 7, the rheological characteristics (loss modulus, storage modulus, viscosity) and sol–gel transition temperature were determined.

### 4.3. Method for Measuring the Loss Modulus, Storage Modulus, and Gelation Temperature

To determine the temperature at which gelation occurs, a temperature change test from 10 to 40 °C was performed using a SmartPave 102e rheometer (Anton Paar, Graz, Austria). Samples with a volume of 1 mL were placed in an ultrasonic bath (Stegler, Moscow, Russia) and treated for 10 min to remove visible air bubbles. Then, the samples were applied to the rheometer plane. The measurement was carried out on a CP50-1 cone-plane measuring system (diameter 50 mm, angle 1°) at a frequency of 1 Hz, a shear strain of 1%, and a gap of 0.1 mm. The heating rate of the plane was 0.05 °C/sec. The storage moduli (G′) and loss moduli (G″), representing elastic energy storage (well-structured state) and viscous energy dissipation (viscous state), respectively, were recorded during heating of the samples [69]. The transition from a viscous liquid state (G′  <  G″) to an elastic semi-solid state (G′  > G″) was determined according to the change in the values of G′ and G″. The gelation temperature (T_sol–gel_) was determined as the intersection point of the storage and loss moduli (G′ = G″). The measurement was performed in triplicate for each sample.

### 4.4. Viscosity Measurement Technique

The viscosity of the nasal thermosensitive in situ gels was measured using a SmartPave 102e rheometer (Anton Paar, Austria) at room temperature (20 °C). A CP50-1 cone-plate measuring system (50 mm diameter, 1° angle) was used to determine the viscosity at a shear rate of 1 sec^−1^ and a gap of 0.1 mm. The average values of 300 points were used to calculate viscosity. Triplicate measurements were performed for each sample.

### 4.5. Density Measurement Technique

The density of nasal thermosensitive gels was determined in situ using a VIP-2MR vibratory density meter (Termex, Saint Petersburg, Russia). Two milliliters of hydrogel were introduced into a measuring cell thermostatted at 20 °C. Three replicates were run for each sample.

### 4.6. SprayVIEW Methodology

**Figure 9:**
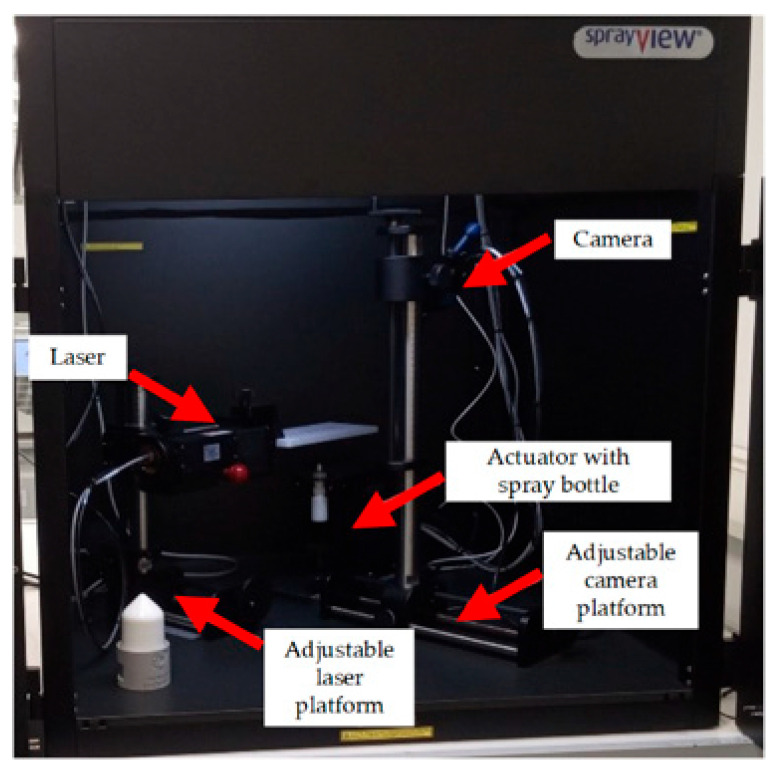
External appearance of the SprayVIEW measuring system.

The spray angle of nasal thermosensitive in situ gels was assessed using the SprayVIEW measuring system (Proveris Scientific, Hudson, MA, USA) (Figure 9).

The settings of the SprayVIEW measuring device were selected empirically, taking into account the need to obtain an image that could be processed by Viota software (Proveris Scientific, USA).

The selected instrument distances were as follows: horizontal camera position 23.0 cm; vertical camera position 14.0 cm; lens aperture 2.0; horizontal laser position 4.0 cm; vertical laser position 14.0 cm; laser depth 5.5 cm; actuator position −0.5 cm; and orientation of the device’s diaphragm tip relative to the laser beam 0°. The center of the coordinate system on the screen in the software window was set to the center of the dispensing device tip.

To ensure proper operation of the device, a calibration procedure was performed before the measurements. Before and after each spray, droplets of the in situ temperature-sensitive gels were removed from the tip of the dispensing device. The vial containing the dispensing device was placed in a special holder, which was installed on top of the actuator. To measure the SprayVIEW spray pattern geometry, a method was used that included characterization and three single activations for each sample.

### 4.7. Mathematical Modeling of Spray Torch

The application of mathematical modeling methods in this article aimed at studying the influence of the characteristics of the compositions of nasal formulations on the spray angle and subsequent comparison of the obtained results with the SprayVIEW system to assess the adequacy of the mathematical model.

To predict the spray angle of nasal formulations, the discrete particle method (DPM) was used in this study. In the DPM method, the trajectories of drug particles or droplets are calculated individually at specified intervals while calculating the continuous phase, whose motion is determined by solving the Navier–Stokes equations. The dispersed phase (droplets) can exchange momentum, mass, and energy with the continuous phase.

This paper examined a multiphase problem. The system consists of a continuous medium, represented by an air flow, and a dispersed phase, represented by particles (droplets), in a non-stationary state. The mathematical description for the continuous medium is based on the equations of conservation of mass (continuity) and momentum; heat transfer is not considered in the model:

(2) $$\frac{\partial \rho \alpha }{\partial t}+\nabla \cdot \left(\alpha \rho \vec{v}\right)=0,$$

(3) $$\frac{\left.\partial (\alpha \rho \vec{v}\right)}{\partial t}+\nabla \cdot \left(\alpha \rho \vec{v}\vec{v}\right)=-\alpha \nabla P+\nabla \left(\alpha \overset{̿}{\tau }\right)+\alpha \rho \vec{g}+{\vec{R}}_{sl},$$

(4) $${\vec{R}}_{sl}=\frac{1}{∆V}\sum {\vec{f}}_{p,i},$$

(5) $${\vec{f}}_{p}=\frac{V_{p}K_{sl}}{\alpha_{p}}\left(\vec{v}-{\vec{v}}_{p}\right),$$

were *α* and *α_p_* are the fractions of the continuous medium and the dispersed phase; *t* is time, s; ${\vec{R}}_{sl}$ is the force characterizing the influence of the dispersed phase on the continuous medium, kg/m^2^‧s^2^; ${\vec{f}}_{p}$ is the force characterizing the resistance of the particle, N; *i* is the particle index; *V_p_* is the particle volume, m^3^; *K_sl_* is the momentum exchange between the phases, kg/(m^3^‧s); ${\vec{v}}_{p}$ is the particle velocity vector, m/s.

To resolve the fundamental equations of the mathematical model (2–5), additional relationships are required. Since the flow is considered a viscous compressible flow, the system must be supplemented with an equation of state describing the dependence of the flow density on pressure (6) and an equation defining the viscous stress tensor (7):

(6) $$\overset{̿}{\tau }=\mu \left(\left(\nabla \vec{v}-\nabla {\vec{v}}^{T}\right)-\frac{2}{3}\vec{v}\cdot I\right),$$

(7) P=ρRT,

were $\overset{̿}{\tau }$ is the viscous stress tensor; *μ* is the dynamic viscosity, Pa‧s; $\vec{v}$ is the velocity vector of the continuous medium, m/s; *I* is the unit tensor; *P* is the gas pressure, Pa; ρ is the density of the continuous medium, kg/m^3^; *R* is the universal gas constant, J/(mol‧K); *T* is the gas temperature, K.

To describe the movement of the dispersed phase, the following equation is used:

(8) $$m_{p}\frac{d{\vec{v}}_{p}}{dt}=m_{p}\vec{g}+{\vec{f}}_{p,c}+{\vec{f}}_{p,w},$$

In this case, the interaction between the particle and the continuous medium is described by the following equations:

(9) $${\vec{f}}_{p,c}={\vec{f}}_{p}+{\vec{f}}_{\nabla p}+{\vec{f}}_{\nabla \tau },$$

(10) $${\vec{f}}_{\nabla p}=-V_{p}\nabla p,$$

(11) $${\vec{f}}_{\nabla \tau }=-V_{p}\nabla \cdot \overset{̿}{\tau },$$

were *m_p_* is the particle mass, kg; $\vec{g}$ is the acceleration due to gravity, m/s^2^; ${\vec{f}}_{p,c}$ is the interaction force between the continuous medium and the particle, N; ${\vec{f}}_{p,w}$ is the interaction force between the particle and the wall, N; ${\vec{f}}_{\nabla p}$ is the pressure gradient force acting on the particle, N; ∇*p* is the pressure gradient, Pa; ${\vec{f}}_{\nabla \tau }$ is the viscous force, N.

The given system of equations is solved under the following initial and boundary conditions for the continuous and dispersed phases:

(12) $$\vec{v}{\left(x,y,t\right)}_{t=0}={\vec{v}}_{init},$$

(13) $${\vec{v}}_{p}{\left(x,y,t\right)}_{t=0}={\vec{v}}_{p,init},$$

(14) $$\vec{v}(x_{inlet},y_{inlet},t)={\vec{v}}_{0},$$

(15) $$\vec{v}(x_{wall},y_{wall},t)=0,$$

(16) $${\vec{v}}_{p}(x_{inlet},y_{inlet},t)={\vec{v}}_{p,0},$$

(17) $${\vec{v}}_{1,init}={\vec{v}}_{2,init},$$

were ${\vec{v}}_{1,init}$ and ${\vec{v}}_{2,init}$—the velocity of the particle before and after collision with the wall, m/s.

The presented system of differential equations was solved using the finite volume method implemented in the ANSYS Fluent 17.0 software package.

Hydraulic-type dispersing devices are most often used for spraying liquid nasal dosage forms. Pressure in these dispersing devices is generated by compressing a piston, and the liquid passes through a narrow orifice and disperses spontaneously. Therefore, when modeling the spray pattern in ANSYS Fluent 17.0, the plain-orifice atomizer model, corresponding to a hydraulic-type dispersing device, was selected using the DPM method. In this model, the liquid passes through a nozzle and accelerates, forming a liquid jet (primary disintegration), and then disintegrates into numerous small droplets (secondary disintegration). This model allows one to calculate the size distribution of liquid droplets and determine their trajectories upon exiting the dispersing device.

**Figure 10:**
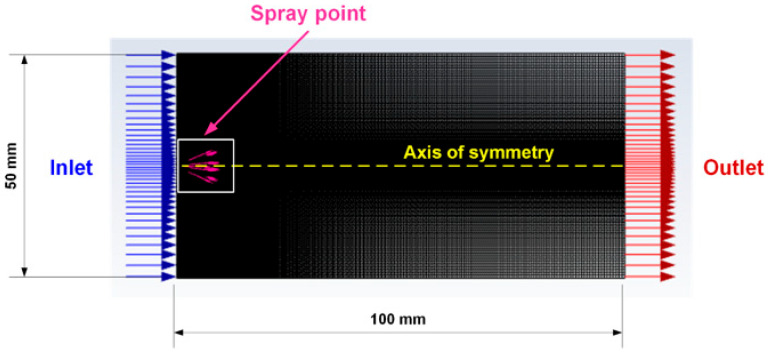
Visualization of the 2D spray zone geometry.

The spray pattern was simulated using 2D geometry, representing a rectangular area with characteristic dimensions of 50 × 100 mm within which the spray occurred. A spray point with a diameter of 0.3 mm was located at the left center of the 2D geometry (the entry area) (Figure 10).

Since the problem is axisymmetric, to simplify the model and reduce the time required to implement one calculation, only half of the 2D geometry was constructed relative to the axis of symmetry, and the second half was reflected after the calculations were completed.

## Figures and Tables

**Table 1:** Gel temperature of the P407-based formulations.

Sample	P407, wt%	Mean Tsol–Gel Value ± SD (°C, *n* = 3)	G′ at 35 °C ± SD (Pa, *n* = 3)
P1	15	32.71 ± 0.47	209.15 ± 23.71
P2	18	26.18 ± 0.81	5501.83 ± 349.47
P3	20	24.63 ± 0.14	8581.36 ± 236.18

**Table 2:** Gel temperature of P407 and P188 based formulations.

Sample	P407 + 188, wt%	P407/P188 Ratio	Mean Tsol–Gel Value ± SD (°C, *n* = 3)	G′ at 35 °C ± SD (Pa, *n* = 3)
PP1	18	9/1	36.47 ± 0.73	4627.12 ± 203.17
PP2	18	8/2	41.73 ± 0.19	3083.94 ± 294.56
PP3	20	9/1	30.28 ± 0.33	7294.35 ± 348.81
PP4	20	8/2	38.31 ± 0.48	5067.62 ± 294.63

**Table 3:** Gel temperature of the P407-based formulations and mucoadhesive polymers.

Sample	P407, wt%	Mucoadhesive Polymer, wt%			Mean Tsol–Gel Value ± SD (°C, *n* = 3)	G′ at 35 °C ± SD (Pa, *n* = 3)
		Chitosan	HPMC	Alg-Na		
PC1	18	0.2	-	-	28.87 ± 0.34	407.37 ± 34.81
PC2	18	0.4			27.36 ± 0.48	465.22 ± 27.93
PC3	18	0.6			25.91 ± 0.69	502.51 ± 36.67
PC4	20	0.2			26.37 ± 0.43	3029.68 ± 321.54
PC5	20	0.4			25.85 ± 0.61	3187.25 ± 298.37
PC6	20	0.6			24.83 ± 0.19	3422.92 ± 309.58
PH1	18	-	0.5		27.45 ± 0.37	5847.13 ± 421.39
PH2	18		1		26.51 ± 0.53	6274.39 ± 481.38
PH3	18		2		26.06 ± 0.64	6691.57 ± 395.86
PH4	20		0.5		25.46 ± 0.38	8838.67 ± 513.55
PH5	20		1		25.17 ± 0.49	8901.29 ± 518.97
PH6	20		2		25.39 ± 0.51	9084.46 ± 497.83
PA1	18		-	0.2	25.43 ± 0.38	6326.84 ± 318.57
PA2	18			0.4	25.45 ± 0.19	6681.63 ± 476.24
PA3	18			0.6	25.49 ± 0.23	7264.91 ± 539.41
PA4	20			0.2	24.57 ± 0.18	10,125.63 ± 512.85
PA5	20			0.4	24.72 ± 0.36	10,554.82 ± 618.38
PA6	20			0.6	24.40 ± 0.28	11,155.37 ± 695.93

**Table 4:** Density and viscosity values of the in situ thermosensitive nasal gel samples.

Sample	Mean Density ± SD (g/cm^3^, *n* = 3)	Mean Viscosity at 20 °C ± SD (mPa·s, *n* = 3)
P1	1.003 ± 0.004	10.44 ± 0.45
P2	1.006 ± 0.006	18.22 ± 0.46
P3	1.007 ± 0.003	37.71 ± 1.47
PP1	0.994 ± 0.004	14.78 ± 0.67
PP2	0.997 ± 0.005	26.12 ± 0.91
PP3	1.000 ± 0.007	41.16 ± 1.35
PP4	1.003 ± 0.002	24.02 ± 0.74
PC1	1.001 ± 0.003	35.41 ± 2.01
PC2	1.000 ± 0.005	68.13 ± 6.04
PC3	1.006 ± 0.004	170.01 ± 10.89
PC4	0.999 ± 0.006	46.13 ± 3.47
PC5	1.000 ± 0.003	72.75 ± 6.11
PC6	1.004 ± 0.003	201.72 ± 18.29
PH1	1.006 ± 0.007	32.31 ± 2.99
PH2	1.009 ± 0.005	38.90 ± 2.28
PH3	1.011 ± 0.002	48.16 ± 3.76
PH4	1.010 ± 0.006	51.84 ± 5.29
PH5	1.011 ± 0.003	60.83 ± 5.93
PH6	1.014 ± 0.005	71.34 ± 6.19
PA1	1.003 ± 0.004	99.45 ± 7.57
PA2	1.004 ± 0.005	206.03 ± 20.52
PA3	1.007 ± 0.007	457.14 ± 51.19
PA4	1.010 ± 0.002	167.61 ± 18.05
PA5	1.013 ± 0.006	310.40 ± 48.47
PA6	1.017 ± 0.003	573.45 ± 60.12

**Table 5:** Dependence of spray angle on the viscosity of the compositions.

Sample	Mean Viscosity at 20 °C ± SD (mPa·s, *n* = 3)	Mean Spray Angle ± SD (°, *n* = 3)
P2	18.22 ± 0.46	19.30 ± 2.86
PP3	41.16 ± 1.35	14.93 ± 1.62
PC1	35.41 ± 2.01	15.03 ± 2.10
PC2	68.13 ± 6.04	12.6 ± 3.27
PC4	46.13 ± 3.47	13.80 ± 1.23
PH1	32.31 ± 2.99	16.43 ± 2.48
PH2	38.90 ± 2.28	14.63 ± 4.19

**Table 6:** Results of calculating the relative error of the computational experiments.

Sample	Spray Angle, °		*δ*, %
	Experiment	Model	
P2	19.30	20	3.63
PP3	14.93	15	0.47
PC1	15.03	16	6.45
PC2	12.6	12	4.76
PC4	13.80	14	1.45
PH1	16.43	17	3.47
PH2	14.63	16	9.36

**Table 7:** Compositions of the thermosensitive in situ gels.

Sample	P407, wt%	P188, wt%	Chitosan, wt%	HPMC, wt%	Alg-Na, wt%
P1	15	-	-	-	-
P2	18				
P3	20				
PP1	18 (9/1)		-	-	-
PP2	18 (8/2)				
PP3	20 (9/1)				
PP4	20 (8/2)				
PC1	18	-	0.2	–	–
PC2	18		0.4		
PC3	18		0.6		
PC4	20	-	0.2	-	-
PC5	20		0.4		
PC6	20		0.6		
PH1	18	-	-	0.5	-
PH2	18			1	
PH3	18			2	
PH4	20	-	-	0.5	-
PH5	20			1	
PH6	20			2	
PA1	18	-	-	-	0.2
PA2	18				0.4
PA3	18				0.6
PA4	20	-	-	-	0.2
PA5	20				0.4
PA6	20				0.6

## Data Availability

The original contributions presented in this study are included in the article. Further inquiries can be directed to the corresponding authors.

## Abbreviations

The following abbreviations are used in this manuscript:

P407	Poloxamer 407
P188	Poloxamer 188
HPMC	Hydroxypropyl methylcellulose
T_sol–gel_	Gelling temperature
PPO	Poly(propylene oxide)
PEO	Poly(ethylene oxide)
G′	Storage modulus
G″	Loss modulus
SD	Standard deviation
Alg-Na	Sodium alginate
DPM	Discrete particle method
P	P407
PP	P407 + P188
PC	P407 + Chitosan
PH	P407 + HPMC
PA	P407 + Sodium alginate
